# Alzheimer Disease: Failure to Tune Out Irrelevant Input?

**DOI:** 10.1371/journal.pmed.0020356

**Published:** 2005-09-20

Some people close their eyes when they listen to music; others wear earplugs when they read in noisy buses or airplanes. Both serve to block out inappropriate sensory stimulation when we try to focus, something that happens automatically when we concentrate. Neuroimaging studies have shown that this focusing process is regulated within our brains; when people undergo tasks that depend exclusively on visual clues, they not only activate the areas of the brain involved in visual processing but also deactivate those areas that have to do with auditory processing. The reverse activation/deactivation pattern is seen when the clues are exclusively auditory. This phenomenon, called “cross-modal auditory/visual deactivation,” is thought to help focus the information processing capacities of the brain on the relevant areas

Alexander Drzezga and colleagues are trying to understand the cognitive changes associated with Alzheimer disease (AD). AD has historically been categorized as a disease of memory loss, but more recent results suggest that the disease is associated with more fundamental deficits in attention and perception. The researchers now report results from a study that examined the activation and deactivation patterns in the brains of patients with AD. The results suggest that patients with AD and patients with mild cognitive impairment (MCI) have problems focusing their brain activity: they show less activity than normal people in the “correct” brain areas but also more activity in the “wrong” areas.

To test whether cross-modal inhibition is affected in AD, the researchers recruited 32 participants, who fell into three age-matched groups: 11 healthy individuals, 11 individuals with MCI, and ten individuals with moderate AD. All participants were trained to perform a navigation task, based exclusively on visual clues, in a virtual reality environment. All participants understood the task and the clues. They then each completed 12 separate positron emission tomography scans, eight during active navigation (finding their way from a starting point to a specific destination) and four under control conditions (traveling along a never-ending pathway). The tasks were performed in complete silence.

The three groups differed in their performance—measured by the time needed to reach the destination—as would be expected. In addition, the researchers observed differences in their brain activation and deactivation patterns. Higher-order visual processing areas were activated to the greatest extent in healthy individuals, to a lesser extent in individuals with MCI, and not at all in individuals with AD. Similarly, cerebellar activation (suggesting movement automation) was absent in individuals with AD, present at low levels in individuals with MCI, and strongest in healthy individuals. Conversely, individuals with AD or MCI showed more activation in the (lower order) primary visual areas and in frontal cortical areas. This may indicate that sensory information gets “stuck” in lower levels of processing in AD. Regarding the inhibition of irrelevant input, strong bilateral deactivation in task-irrelevant auditory cortical regions was seen in healthy individuals. This was much less prominent in individuals with MCI, and individuals with AD showed no deactivation. [Fig pmed-0020356-g001]

**Figure pmed-0020356-g001:**
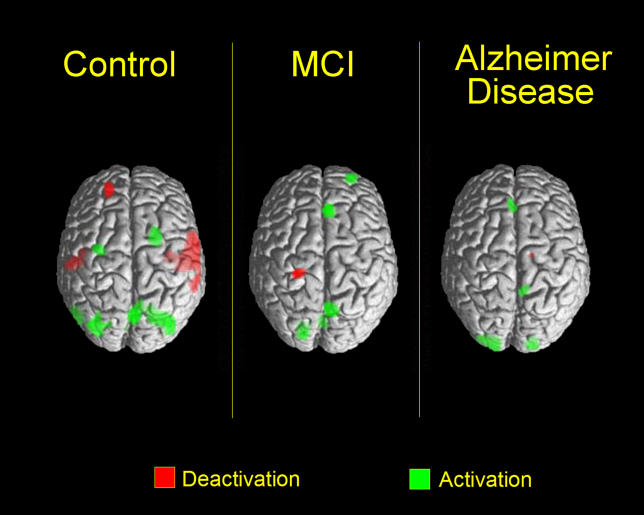
Cerebral activation and deactivation during spatial navigation

The researchers conclude that “successful cognitive performance in healthy individuals is associated with deactivation of task-irrelevant cerebral regions, whereas the development of AD appears to be characterized by a progressive impairment of cross-modal cerebral deactivation functions.” They go on to propose that “orientation disability in the outside world seen in patients with AD may in fact be partially based on the inability to selectively orient spatial attention to task-relevant internal representations of perceptual stimuli.” The researchers are now in the middle of a follow-up study that concentrates on individuals with MCI—some of whom remain stable for years, whereas others progress to AD—to see whether the extent of cross-modal inhibition correlates with progression to disease.

